# Poor performance of the rapid test for human brucellosis in health facilities in Kenya

**DOI:** 10.1371/journal.pntd.0005508

**Published:** 2017-04-07

**Authors:** William A. de Glanville, Raquel Conde-Álvarez, Ignacio Moriyón, John Njeru, Ramón Díaz, Elizabeth A. J. Cook, Matilda Morin, Barend M. de C. Bronsvoort, Lian F. Thomas, Samuel Kariuki, Eric M. Fèvre

**Affiliations:** 1 Centre for Immunity, Infection and Evolution, Institute for Immunology and Infection Research, School of Biological Sciences, University of Edinburgh, Edinburgh, United Kingdom; 2 International Livestock Research Institute, Nairobi, Kenya; 3 Institute for Tropical Health, Navarra Institute for Sanitary Research and Department of Microbiology and Parasitology, Medical School, University of Navarra, Pamplona, Spain; 4 Centre for Microbiology Research, Kenya Medical Research Institute, Nairobi, Kenya; 5 Institute for Bacterial Infections and Zoonoses, Friedrich Loeffler Institute, Berlin, Germany; 6 Department of Medical Biochemistry and Microbiology, Uppsala University, Uppsala, Sweden; 7 Epidemiology, Economics and Risk Assessment Group, The Roslin Institute, University of Edinburgh, Edinburgh, United Kingdom; 8 Institute of Infection and Global Health, University of Liverpool, Liverpool, United Kingdom; Swiss Tropical and Public Health Institute, SWITZERLAND

## Abstract

Human brucellosis is considered to be an important but typically under-diagnosed cause of febrile illness in many low and middle-income countries. In Kenya, and throughout East Africa, laboratory diagnosis for the disease is based primarily on the febrile antigen *Brucella* agglutination test (FBAT), yet few studies of the diagnostic accuracy of this test exist. Assessment of the performance of the FBAT is essential for its appropriate clinical use, as well as for evaluating surveillance data reported by public health systems. To assess FBAT performance, we collected sera from people with symptoms compatible with brucellosis attending two health facilities in Busia County, Kenya. Sera were tested using the FBAT and results compared with those from the Rose Bengal Test (RBT), an assay with well-known performance characteristics. Positives on either test were confirmed using the classical serum agglutination test (SAT)-Coombs test combination and a rapid IgM/IgG lateral flow immunochromatography assay (LFA). A questionnaire focussing on known risk factors for exposure to *Brucella* spp. was also conducted, and relationships with FBAT positivity examined using logistic regression. Out of 825 recruited individuals, 162 (19.6%) were classified as positive using the FBAT. In contrast, only eight (1.0%) were positive using the RBT. Of the 162 FBAT positives, one (0.62%) had an atypical agglutination in SAT and three (1.9%) showed low Coombs titres. Out of 148 FBAT positive individuals tested using the LFA, five (3.4%) were IgM positive and none were IgG positive. Poor or no correlation was observed between FBAT results and most established risk factors for *Brucella* infection. We observed substantial disagreement between the FBAT and a number of well-known serological tests, with the majority of reactive FBAT results appearing to be false positives. Poor FBAT specificity, combined with a lack of confirmatory testing, strongly suggests overdiagnosis of brucellosis is common in this low prevalence setting. This is expected to have important economic impacts on affected patients subjected to the long and likely unnecessary courses of multiple antibiotics required for treatment of the disease.

## Author summary

Brucellosis is a debilitating disease of people caused by infection with one of a number of different *Brucella* species. In almost all cases, people acquire the infection through exposure to infected animals or contaminated animal products. Human brucellosis is well known for its wide range of symptoms, and is often clinically indistinguishable from other infectious diseases, such as malaria or typhoid. Diagnosing the disease therefore typically relies on laboratory tests. A wide range of tests are available, but little is known about the accuracy of the principal test used in Government health facilities in Kenya, the febrile *Brucella* agglutination test (FBAT). In this study, we identified people with symptoms compatible with brucellosis attending health centres in Kenya. By comparing results from the FBAT performed on samples collected from these individuals with the results from a range of well-established diagnostic tests, we were able to show that the FBAT produces large numbers of false positive results. We expect that this leads to a high levels of overdiagnosis of brucellosis in some parts of Kenya. Treatment of the disease involves multiple weeks of multiple antibiotics, and these incorrect diagnoses may have important and unnecessary negative impacts on affected patients.

## Introduction

Brucellosis is a zoonotic disease with a global distribution that can cause severe illness in people and important economic losses in livestock [1–4]. *Brucella abortus* and *B*. *melitensis* are the most important zoonotic pathogens, and both species are likely to be endemic in cattle and small ruminants in Kenya [5–7].

In humans, brucellosis is a debilitating disease that can cause a wide range of symptoms including fever, arthralgia, myalgia and fatigue [8–10]. Development of focal (e.g. joint, pulmonary, gastrointestinal, hepatobiliary, genitourinary and neurological) complications is common and influenced by the length of time before diagnosis and initiation of treatment [11,12]. Treatment of human disease requires long courses of combined antibiotics and whilst rates of relapse are low with the best regimes [13], compliance in resource-limited countries is often difficult to achieve. Brucellosis can therefore be a considerable economic burden on affected individuals, both in terms of the cost of therapy and days of potential work lost [14,15]. Whilst animal contact is a major risk factor for human infection, *Brucella* spp. are excreted in milk and may be present in the offal and meat of infected animals [16]. Hence, individuals living in both livestock and non-livestock keeping households may be at risk in areas in which *Brucella* spp. are endemic in animals. There is no human vaccine, and prevention of infection relies on reducing people’s exposure to infected animals and animal products [16].

Little is known about the prevalence of human brucellosis in sub-Saharan Africa [1,17]. The disease can mimic a variety of acute febrile illnesses, and laboratory tests are essential for diagnosis. Identifying cases presents a particular challenge in settings with limited laboratory capacity and where better-known causes of fever, such as malaria or typhoid, co-occur [1,17–21]. It is generally considered that human brucellosis is under-diagnosed in many of the areas in which it is endemic in livestock [9,22,23].

Culture of clinical specimens has 100% specificity, but often poor sensitivity, particularly in chronic cases, and also requires the availability of appropriate facilities [24]. Serological tests are therefore the mainstay of laboratory diagnosis in the majority of endemic areas. Serum agglutination assays using whole smooth *Brucella* cell suspensions as antigens are the most commonly used test and are available in a variety of formats. These include the rapid slide *Brucella* agglutination test performed with plain serum at neutral pH and the buffered plate agglutination tests, a family of rapid tests that use *B*. *abortus* suspensions in lactate buffer at low pH. The Rose Bengal Test (RBT) is the most widely used of the buffered plate agglutination tests [25]. While these rapid tests may be used to provide a semi-quantitative assessment of antibody titre through serial dilutions, the standard tube agglutination test (SAT), performed with serum dilutions in tubes or microplates at neutral pH using saline as the diluent, enables more accurate assessment of antibody levels [24]. Infection with *Brucella* can result in the production of antibodies that do not agglutinate at neutral pH, which steadily increase and replace agglutinating antibodies over the course of the infection [11,25–27]. Thus, the SAT is usually complemented with the *Brucella* Coombs test [28], an IgG and IgA anti-immunoglobulin assay [11,24,25]. All of these tests detect antibodies to the *Brucella* smooth lipopolysaccharide [24], and the purified antigen is also used in a variety of non-agglutination based tests, including the lateral flow immunochromatographic assay (LFA) [29,30]. The sensitivity and specificity of serological testing for brucellosis varies depending upon the characteristics of the test and the antigenic suspensions used, as well as the stage of infection [24–27]. Reduced specificity can result from the persistence of anti-lipopolysaccharide antibody titres in a proportion of recovered patients as well as infections by cross-reacting bacteria [26,27,31–33].

The serological test currently used for the laboratory diagnosis of human brucellosis in government health facilities in Kenya and, to our knowledge, throughout Tanzania and Uganda, is a variant of the rapid slide *Brucella* agglutination test. There are several commercial kits currently available in East Africa, comprising separate *‘melitensis’* and *‘abortus’* antigens, marketed as part of a "febrile antigen test” kit. Despite their widespread use in East Africa, very little has been reported regarding the diagnostic accuracy of these febrile antigen *Brucella* agglutination tests (FBAT) or their predictive value in brucellosis endemic areas. Clearly, filling this information gap has important implications for the clinical application of this test. Furthermore, a recent review highlighted the paucity of data available to estimate global human brucellosis burden, and identified the strengthening of public health systems as an important mechanism to improve the quality of data captured through routine reporting [34]. A major consideration in interpreting the data generated by public health systems is the performance of tests used to diagnose brucellosis in endemic areas.

The aim of this study was to assess the performance of the FBAT as currently used in health facilities in Kenya. We conducted a serological and questionnaire-based survey of individuals presenting to health facilities in western Kenya with symptoms compatible with brucellosis. The performance of the FBAT was compared with that of a range of serological assays with well-established diagnostic performance. To provide epidemiological evidence to support test performance, logistic regression was used to explore predictors of FBAT seropositivity.

## Methods

### Ethics statement

Ethical approval for the study was granted by the Kenya Medical Research Institute (KEMRI) ethical review board (SCC1701). All participants provided written informed consent; minors between 5 and 17 signed an assent document and their guardians provided consent.

### Study area

The study was conducted in Busia county which has a total area of 1637 km^2^ and a population of 786,365 people (Open Kenya). Two health facilities were selected for this study. These were Busia County Referral Hospital (BCH) and a private clinic at the Kenya Medical Research Institute (KEMRI) field station at Alupe. Livestock production in the County is characterised by a predominantly small-scale, mixed crop and livestock system [35].

### Patient selection and sampling

Sampling took place on weekdays from June to December 2012. A separate focus of the study was to use epidemiological tools to identify the source of infection in suspect brucellosis cases, with sampling effort intended to maximise the number of identified cases within the study period. Patients attending the outpatient clinic of BCH and the KEMRI clinic were recruited into the study by non-study health centre clinicians. The criterion for inclusion was clinical suspicion of brucellosis, including exposure history and compatible symptoms (see Table 1). We therefore aimed to include those patients who would normally present for laboratory-based brucellosis testing within either health facility. Exclusion criteria were patients less than 5 years of age, minors (5–17 years) not accompanied by a guardian, patients presenting with severe illness requiring hospitalisation, and those patients with an already established diagnosis explaining illness. Patients typically pay for brucellosis diagnostics in government facilities in Kenya (1.50–3.00 USD), but testing was provided free of charge to all participants enrolled in this study.

**Table 1:** Presenting symptoms and exposure characteristics of study participants.

Presenting signs	% (95% CI)	Exposures*	% (95% CI)
Malaise	88.5 (85.8–90.7)	Male gender	31.3 (27.9–34.9)
Headache	79.2 (76.0–82.1)	Travel outside home county	20.4 (17.5–23.6)
Fever	64.6 (60.9–68.1)	Previous brucellosis diagnosis	11.6 (9.3–14.2)
Abdominal pain	60.5 (56.7–64.1)	Know someone with similar symptoms	7.3 (5.5–9.5)
Anorexia	54.3 (50.6–58.1)	Know someone with brucellosis diagnosis	20.3 (17.5–23.5)
Joint pain	51.1 (47.3–54.8)	High risk milk consumption	7.6 (5.8–9.9)
Back pain	43.2 (39.6–47.0)	High risk yoghurt consumption	15.1 (12.5–18.1)
Sweats	42.2 (38.6–46.0)	High risk meat consumption	2.7 (1.6–4.3)
Chills	37.6 (34.0–41.3)	Blood consumption	4.1 (2.8–5.9)
Chest pain	34.4 (30.9–38.1)	Keep cattle at home	47.5 (43.7–51.3)
Muscle aches	34.2 (30.7–37.8)	Keep sheep or goats at home	31.5 (28.1–35.1)
Cough	27.3 (24.1–30.8)	Keep pigs at home	20.6 (17.7–23.8)
Constipation	25.3 (22.2–28.7)	Cattle born in compound	13.6 (11.2–16.4)
Neck pain	17.2 (14.5–20.3)	Sheep/goats born in compound	7.0 (5.3–9.2)
Diarrhoea	12.4 (10.1–15.1)	Direct contact with animal birth products	2.7 (1.6–4.3)
Vomiting	11.1 (8.9–13.7)	Abortion in compound	1.9 (1.0–3.2)
Sore throat	9.2 (7.3–11.7)	Involvement in animal slaughter	1.0 (0.44–2.1)
Breathlessness	6.8 (5.1–9.0)		
Weight loss	3.2 (2.0–4.8)	Any high risk food consumption	21.4 (18.4–24.6)
Joint swelling	3.0 (1.9–4.6)	Any high risk animal contact	5.5 (3.9–7.6)
Rash	2.3 (1.4–3.8)	Any high risk activity (food or animal)	25.8 (22.6–29.4)
Orchitis (in males)	0.9 (0.2–3.5)		

* In the 180 days prior to onset of clinical symptoms.

A study technician collected 6 ml of blood from study participants into a plain tube. Clotted blood was spun at 3000 rpm for 5 to 10 minutes and serum extracted.

### Patient interviews

Each consenting or assenting participant underwent a questionnaire interview with a study clinician who collected information on clinical history, presenting clinical symptoms, and health seeking behaviour. On the basis of a maximum expected brucellosis incubation period of 6 months [36], participants were given a structured questionnaire in which they were asked to recall high-risk exposures (i.e. to food products or livestock) in the 180 days prior to the onset of symptoms.

### Serological assays in hospital

Serum was used to perform the FBAT and RBT at BCH and the KEMRI clinic. Antigens for the FBAT used in these settings were manufactured by Fortress diagnostics, UK (http://www.fortressdiagnostics.com, "Febrile Antigen Kit") and purchased locally. Standard hospital operating procedures were used for the FBAT. These were the same in both health centres and involved mixing a 50μl drop of serum with a drop (approximately 50μl) of ‘*abortus*’ antigen and drop of ‘*melitensis*’ antigen on a dedicated agglutination slide. Serum and antigen solution were rotated on a mechanical rotator for 2 minutes. As per standard procedures in both health facilities, tests were reported as ‘reactive’ if any evidence of agglutination was observed on naked eye examination. While the study health centres did not routinely measure antibody titres in reactive samples, these were semi-quantitatively assessed as part of this study using a serial dilution approach as described by the manufacturers. For this, 80, 40, 20, 10 and 5 μl drops of serum were placed on agglutination tiles and a 50 μl drop of reagent was added to each, followed by mixing and rotation for 2 minutes. Agglutination in the first, second, third, fourth or fifth well was considered suggestive of a 1:20, 1:40, 1:80, 1:160 or 1:320 titre, respectively.

The RBT performed in each health centre followed standard procedures [25] and involved mixing 25μl of serum with 25μl of antigen on a white glossy ceramic tile with a wooden toothpick and rotation on a mechanical rotator for 4 minutes. The antigen, produced at the University of Navarra, was a suspension of fully smooth *B*. *abortus* 1119 standardized according to internationally established guidelines [37] and controlled for quality using a panel of brucellosis positive and negative serum samples [38]. Samples were confirmed as reactive if any perceptible agglutination, including the formation of a ring around the sample [25], was observed on naked eye examination.

Sera that were reactive using either the RBT or FBAT was subjected to the rapid LFA for the presence of IgG and IgM antibodies [30]. This test was marketed by Life Assay (South Africa) and was performed according to the manufacturer’s instructions.

Remaining serum was stored at -40°C on the day of collection before being shipped on ice to the University of Navarra, Spain, for confirmatory serological testing. All samples were shipped at the end of the sampling period.

### Confirmatory testing

At the University of Navarra, samples from the 825 individuals were subjected to a repeat of the RBT. The second RBT was performed by mixing 30μl of serum with 30μl of the aforementioned antigen on a white glossy tile, followed by gentle rotation by hand for 4 minutes, and sera yielding doubtful results rotated for an additional period of 4 minutes. Positive samples were subjected to semi-quantitative titration using the modified version of the test [25]. For this, eight 30μl drops of saline were dispensed on the tile and the first mixed with 30 μl serum. From this, 30μl was transferred to the second drop using a micropipette. This was repeated for each drop to derive dilutions ranging from 1:2 to 1:64. Each drop was tested with an equal volume (30 μl) of the RBT reagent, resulting in a range of final dilutions from 1:2 to 1:128. Positive samples on the FBAT or RBT were also subjected to the SAT and Coombs test. The SAT was performed in a microplate format using a standardised antigenic suspension of *B*. *abortus* 1119–3 as previously described [39] and serum dilutions from 1:20 to 1:2,560. The Coombs test was performed with anti-total human immunoglobulin serum in microplates [40]. A titre ≥1:160 was considered suspicious for the SAT. The Coombs test was considered positive for a titre ≥ 2 times the SAT titre from the same sample [24].

A random selection of FBAT positive samples (n = 24) were retested by technicians at the University of Navarra using the same FBAT test procedure performed in participating health centres, but with a different kit (Febrile Serodiagnostics, Biosystems).

### Data analysis

Multivariable logistic regression was used to assess the relationship between known risk factors for brucellosis and the log odds of seropositivity to the FBAT based on a 1:2 dilution (i.e. equal volumes of serum and each antigen). Risk factors were extracted from the patient interview and were age in years, sex, consumption of high risk milk and yoghurt products, consumption of high risk meat or blood products, direct contact with the birth products of livestock (cattle, sheep, goats or pigs), abortion of livestock in the participant’s compound, involvement in livestock slaughter, and knowing someone with a brucellosis diagnosis in the 6 months prior to the onset of symptoms. Food exposures were considered ‘high risk’ when products were consumed without prior heat treatment, or without adequate heat treatment in the case of blood and meat. The number of variables included in the final model was determined based on the number of FBAT positive results. One variable was selected for every 10 positive cases in order to ensure model complexity did not exceed the available number of degrees of freedom [41]. To meet this criterion, variables for inclusion were either a summarised ‘high risk’ exposure to any food or livestock, or the component parts (i.e. different food types or different livestock exposure types) where the sample size of FBAT cases allowed. No further model selection was performed. Model fit was assessed using the le Cessie-van Houwelingen normal test. Analysis was conducted using the *rms* package [41] in R, version 3.1.1. (http://cran.r-project.org/).

## Results

### Participant enrolment and demographics

A total of 825 individuals attending our study sites for care were recruited into the study, with 691 (84%) coming from BCH and 134 (16%) from the KEMRI clinic. It was possible to conduct the patient survey and collect clinical data from 703 (85%) of these. The presenting symptoms and potential *Brucella* spp. exposure characteristics are summarised in Table 1. The average age of participants was 37.6 years, with a median of 35 and a range between 5 and 98 years. The mean duration of symptoms was 118 days, but this was skewed by a small number of individuals (n = 8) who reported the duration of current illness as more than 3 years. The median value was 14 days. The majority of individuals (426, 60.6%) reported that this was their first visit to a health care provider for their current illness. Of the remaining 277, 177 (64%) had already visited one health facility without resolution, 61 (22.2%) had visited two, 20 (7.2%) had visited three, 11 (3.9%) had visited four and eight (2.9%) had visited five or more. The median duration of symptoms in those presenting for the first time was seven days compared to 61 days in those who had previously sought health care for their current condition (p<0.001). High-risk food consumption was reported in 150 (21.4%) participants, and high-risk animal contact (defined as direct contact with animal birth products, animal abortion in the participant’s home compound or involvement in livestock slaughter) in 37 (5.5%) individuals (Table 1).

### Brucellosis sero-diagnostics

On the basis of tests performed within study health facilities, a total of 162 out of 825 (19.6%, 95% CI 17.0–22.5) individuals had reactive FBAT tests (Table 2). At BCH, 147 (21.3%, 95% CI 18.3–24.6) FBAT tests were reactive, while at the KEMRI clinic, 15 (11.2%, 95% CI 6.6–18.0)) were reactive. When the test was performed on serum dilutions, fifteen out of 162 (1.8%, 95% CI 1.1–3.1)) were seropositive at titres ≥1:160 while 2 (0.2%, 95% CI 0.04–0.9) were seropositive at titres ≥1:320, representing 9.3% and 1.2% of all FBAT positive tests, respectively. Of all 162 FBAT positives, 104 (62.4%) were reactive to both the ‘*abortus*’ and ‘*melitensis*’ antigen, while 44 (27.2%) were reactive to the ‘*abortus’* antigen only and 14 (8.6%) reactive to the ‘*melitensis’* antigen only. Of the 15 FBAT tests seropositive at titres ≥1:160, all but 1 were reactive to both *abortus* and *melitensis* antigen, while both of the FBAT tests seropositive at titres ≥1:320 were reactive to both antigens. Individuals with FBAT titres ≥1:160 were more likely to be reactive to both antigens compared to individuals with FBAT titres <1:160 (OR = 8.9, 95% CI 1.3–387, p = 0.01).

**Table 2:** Comparison of FBAT, RBT, SAT Coombs IgG and LFA in the 825 sera.

FBAT (n)		Number of sera					
			Positive in:				
			RBT > 1/4	SAT	Coombs IgG ^a^	LFA IgM	LFA IgG
Positive (162)	RBT+ ^**b**^	8	0	1 ^**c**^	2	2 ^e^	0 ^e^
	RBT-	154	0	0	1	3 ^e^	0 ^e^
Negative (663)	RBT+	0	0	0	0	n.d. ^**d**^	n.d. ^**d**^
	RBT-	663	0	n.d. ^**d**^	n.d. ^**d**^	n.d. ^**d**^	n.d. ^**d**^
Total		825	0	1	3	5	0

^a^ A titre ≥ 2 times the SAT titre was considered as positive.

^b^ RBT in health facilities in Kenya and /or at the University of Navarra (see Table 3).

^c^ This serum developed an atypical agglutination and only at a 1:80–1:160 titre.

^d^ n.d., not done.

^e^ Out of 148 positives tested.

Eight out of all 825 individuals (1.0%, 95% CI 0.4–2.0) were found to be positive using the RBT (Table 2). Of these, 6 were positive in the RBT performed in the study health facilities and four had titres ≥1:160 and 1 had a titre ≥1:320 when FBAT was performed on serum dilution. Similarly, 6 individuals were weakly positive on the basis of the repeat RBT at the University of Navarra. There was some discrepancy between the results of the repeat RBT at the University of Navarra and those performed in study health facilities, with 4 out of 6 results matching on both occasions (Table 3). None of the 8 RBT positive individuals was positive at dilutions equal or higher than 1:4 (Table 2). Positive sera with FBAT titres ≥ 1:160 were much more likely to be also RBT positive than sera with FBAT titres ≤ 1:160 (OR = 22.8, 95% CI = 3.8–168.9, p = <0.001). No FBAT negative sera were RBT positive (Table 2).

**Table 3:** Comparative results for test positive on RBT, LFA, SAT and Coombs.

Sera	RBT 1^a^	RBT 2^a^	LFA (IgM)	SAT	Coombs (IgG)	FBAT ‘A’	FBAT ‘M’
1	+	+	-	1:40	1:160	1:160	1:160
2	+	+	+	1:20	1:20	1:160	1:160
3	+	-	-	1:80–160	1:160	1:320	1:160
4	+	+	-	1:20	1:20	1:160	1:80
5	+	-	+	<1:20	<1:20	1:20	<1:20
6	+	+	-	1:20	1:40	1:40	1:80
7	-	+	-	<1:20	1:640	1:160	1:160
8	-	+	-	1:20	1:20	1:80	<1:20
9	-	-	+	nd	nd	1:160	1:80
10	-	-	+	nd	nd	1:80	1:160
11	-	-	+	nd	nd	1:40	1:20
12	-	-	-	1:20	1:320	1:40	1:40

^a^ RBT 1 was performed in health facilities in Kenya; RBT 2 was performed at the University of Navarra.

Out of the 162 FBAT positive sera tested using the SAT at the University of Navarra, 161 were clearly negative with titres below 1:40. The remaining serum (0.62%) showed a 1:80–1:160 titre (Table 2) which could be considered as suspicious. However, agglutination was atypical and consisted of mucoid filamentous aggregates rather than the typical clumps. This same serum was RBT negative and Coombs negative when tested at the University of Navarra, but was RBT positive when tested in study health facilities. Three (1.4%) sera, all with clearly negative SAT titres (≤ 1:40), were weakly positive using the Coombs test (Table 3), and of these three, two were weakly RBT positive (Tables 2 and 3). A total of 148 of the 162 FBAT reactive sera were tested using the LFA, of which five (3.4%) were also positive using the IgM LFA but negative in the IgG LFA (Tables 2 and 3). Three of these five were positive at FBAT titres ≥1:160 (Table 3).

Of the 24 randomly selected FBAT positive results that were retested at the University of Navarra using a different febrile antigen kit, 23 (96%) were positive.

### Risk factors for FBAT seropositivity

The large number of FBAT seropositives (138 with linked questionnaire data) provided sufficient sample size to allow us to explore the full range of potential predictors of seropositivity. Observed relationships are presented in Table 4. Knowing someone with a brucellosis diagnosis increased the odds of FBAT seropositivity, as did involvement in animal slaughter. Males were at lower risk of FBAT positivity. The goodness-of-fit test indicated no evidence of any lack of model fit (p = 0.2).

**Table 4:** Results of the multivariable analysis using FBAT seropositivity as the outcome.

Predictor	OR (95% CI)	p-value
Age (in years)	1.01 (0.998–1.02)	0.11
Male gender	0.58 (0.36–0.92)	0.02
High risk milk consumption	0.46 (0.17–1.21)	0.12
High risk yoghurt consumption	1.00 (0.56–1.80)	0.99
High risk meat consumption	1.64 (0.55–4.88)	0.38
High risk blood consumption	1.21 (0.46–3.18)	0.70
Contact with livestock birth products	0.93 (0.25–3.45)	0.92
Contact with abortion materials	1.10 (0.29–4.21)	0.89
Involvement in animal slaughter	5.02 (1.04–24.26)	0.04
Knowing someone with brucellosis diagnosis	1.59 (1.01–2.5)	0.04

## Discussion

We have demonstrated substantial disagreement between results from the FBAT and results from a range of serological tests that have been repeatedly shown to have high diagnostic accuracy for human brucellosis [11,24,25,27,30,42]. Whilst brucellosis should not be excluded as a possible cause of febrile illness in the population under study, our results suggest it is an uncommon cause, and certainly much rarer than results from the FBAT would suggest. It is important to stress that the small number of positive results obtained using the RBT, SAT-Coombs combination and LFA are not by themselves indicative of active brucellosis. There is no established diagnostic titre for a single SAT, which varies from 1:80 to 320 depending on levels of endemicity [24], and any SAT result should be considered along with the Coomb's result. In this study, all but one FBAT positive individual had SAT titres < 1:160, and only three had weak Coombs titres. In addition, none of the sera were positive on the RBT at dilutions higher than 1:2, which may suggest previous exposure rather than current infection [25]. Five FBAT positive sera were IgM positive in the LFA. The presence of the rheumatoid factor (a group of auto-antibodies that recognize Fc regions of IgG [43]) is a known cause of false positives in this test [42]. Since IgM is readily detected using the SAT [24], this is a possible explanation for the observed discord between LFA and SAT results. In the absence of bacteriological evidence or the availability of convalescent antibody titres, the few positive sera identified using SAT-Coombs, RBT and LFA can therefore only be considered as "suspicious" for brucellosis. This fact, combined with the low number of “suspicious” cases identified, limits our ability to derive formal estimates of test performance for the FBAT. However, results from this study strongly suggest that this test generates large numbers of false positives and has poor diagnostic specificity. This interpretation is further supported by the fact that we found no evidence of an association between FBAT seropositivity and most of the anticipated high-risk food or animal exposures. The only exception was involvement with animal slaughter, which has been shown to be a risk factor for human brucellosis in other studies [44].

The poor performance of the FBAT cannot be attributed to the particular kit used in health facilities involved in this study, since a high rate of false positive results were also observed at the University of Navarra with a different kit. Neither health facility involved in this study routinely performed serial dilutions for positive cases and, based on discussion with laboratory managers at other health facilities in western Kenya, the semi-quantitative estimation of antibody titres using the FBAT appears to be rare practice in the region. Our ability to evaluate appropriate cut-offs is limited in this study by the absence of confirmed cases and small number of suspicious cases, but there was evidence of a strong association between RBT positivity and titres ≥1:160 on the FBAT, suggesting the use of a higher cut-off may improve the diagnostic specificity of the test.

The low prevalence of brucellosis in the study population combined with the apparently poor specificity of FBAT suggests considerable overdiagnosis of human brucellosis. An important consideration, however, is that the provision of free brucellosis testing to participants may have contributed to the capture of more patients than would typically be tested for the disease in the study health facilities. It is therefore possible that levels of overdiagnosis indicated by this study are exaggerated. In the period from June 2010 to June 2011, BCH (then named Busia District Hospital) recorded 129 cases of brucellosis, whilst we identified 162 individuals as reactive on the FBAT over an approximately 6 month period between June and December 2012. However, given the small number of suspicious cases we observed over the six months of data collection, the number of cases detected in 2010 and 2011 also point to high levels of overdiagnosis for this disease. Typical treatment for brucellosis in health facilities in the study area is 6 weeks of oral doxycycline combined with either 2 weeks of intramuscular streptomycin or 6 weeks of oral rifampicin. Whilst the clinical management of recruited patients was not recorded as part of this study, treatment is routinely recommended in both health facilities to any individual with a reactive FBAT test. We therefore expect that a substantial proportion of brucellosis treatments in western Kenya are inappropriate. This will result in unnecessary economic costs for patients, and may represent important misuse of antibiotics. This is of particular concern for the selection of resistance to rifampicin, a first line treatment for TB [45].

In 2012, 75,256 brucellosis cases in individuals over five years of age were reported to the Kenya Health Information System (www.hiskenya.org), giving an annual incidence of 230 per 100,000 people [46]. We suspect that the vast majority of these cases were diagnosed on the basis of the FBAT. Whilst brucellosis is very likely to be an important disease in Kenya, particularly amongst pastoral communities [5,47], poor performance of the diagnostic test used to derive surveillance data means there is considerable uncertainty regarding the true burden of human brucellosis in the country.

An internet search revealed that all twelve of the FBAT providers identified include both *B*. *abortus* and *B*. *melitensis* suspensions in their kits. The purpose of this is unclear since it was well established in the first half of the 20^th^ century that suspensions of *B*. *abortus* and *B*. *melitensis* can be exchanged for diagnostic purposes [18]. The target for *Brucella* agglutination tests is the O-polysaccharide of the smooth lipopolysaccharide of the cell surface of smooth *Brucella* species, which has a structure dominated by a highly repeated C epitope common to all smooth brucellae [48] (excluding the recently described *B*. *inopinata* BO1 [49]). Hence, there is no serological test that can distinguish infection with *B*. *abortus* versus *B*. *melitensis* [11,18,24,48]. Despite this, we noted a not unsurprising belief amongst laboratory technicians and clinicians working in both study health facilities that the FBAT, marketed with separate *‘abortus’* and *‘melitensis’* antigens, can distinguish between infections with these different *Brucella* species. This misconception is likely to be strengthened by the inconsistent results obtained with the *‘abortus’* and *‘melitensis’* suspensions of FBAT, as illustrated by our work and recently published work from Tanzania [50]. Long and repeated experience [11,18,24,51] shows that the most plausible reasons for these inconsistencies are deficient standardisation of antigen suspensions and poor antigen quality. Smooth brucellae are notorious for their ability to dissociate in vitro, a genetic drift that results in large proportions of rough mutants that lack the O-polysaccharide of the lipopolysaccharide and have profoundly altered cell surface properties [48]. The consequence of this drift is an absence of diagnostically valid epitopes and a strong tendency to autoagglutinate. Such deficient antigenic suspensions are an important cause of false positivity and the likely explanation for the high numbers of false positives obtained with FBAT in this study, as well as for the inconsistency between reactions to ‘*abortus*’ and ‘*melitensis*’ antigens. We consider antigen quality to be a more likely explanation for poor FBAT specificity than cross-reaction with antibodies to other gram negative bacteria, since such cross-reactions are well known to occur for all serological assays that rely on detection of antibodies to the *Brucella* smooth lipopolysaccharide, including RBT, SAT and Coombs [31], and are likely also with the LFA. Whilst the clinical management of infection with *B*. *melitensis or B*. *abortus* is the same, an indirect consequence of the inconsistency between reactions to different FBAT antigens is that results may be misleading with respect to the source of infection, since *B*. *melitensis* is typically associated with small ruminants while *B*. *abortus* is more typically associated with cattle [16].

The catchment area of study health facilities incorporate a mixed farming area with a very high livestock density. Despite this, a substantial proportion of patients presenting for brucellosis diagnostic testing did not report what we considered high risk contacts with livestock or their products over the six months prior to the onset of clinical symptoms. Studies from Tanzania have reported limited awareness of zoonotic disease among health workers [52], including the range of pathways through which *Brucella* species may be transmitted to people [53]. Detailed clinical history taking and the targeting of testing to individuals considered to be at higher risk of brucellosis, is likely to improve the predictive value of serological testing, particularly in low prevalence populations. An important consideration, however, is that whilst we have a broad understanding of the epidemiology of brucellosis in sub-Saharan Africa [1,17], there is generally a lack of information on the specific transmission routes by which people in specific settings acquire their infections. Population-based studies in endemic areas should seek to fill this information gap. Further studies could also seek to derive diagnostic algorithms for human brucellosis that allow for the formalisation of who to test, and who should be considered positive based on a range of criteria, including the results of imperfect serological assays, exposure history and presenting signs [54,55].

Future studies should seek to derive estimates of sensitivity and specificity for the FBAT, including assessment of the value of deriving semi-quantitative estimates of antibody titres. However, given the likely poor specificity we have demonstrated, future work should also examine alternative diagnostic tests that can be used to replace the FBAT in health facilities in western Kenya and the region more widely. The standard RBT is inexpensive and straightforward to perform, follows the same general procedure as the FBAT and thus requires the same basic laboratory equipment and expertise. Several authors have advocated for the routine use of this test in the diagnosis of human brucellosis in health facilities in resource limited settings [25,56,57]. This test showed considerably better agreement with the results of the SAT-Coombs and LFA than the FBAT in this setting, and is likely to represent a better alternative. A potential limitation of the RBT, although one that applies to all rapid agglutination tests, including the FBAT, are inconsistencies between technicians in reading agglutination results [58]. In our study, there was some discrepancy between results of the RBTs performed in Kenya and those performed by different operators in Spain. One of the two weak RBT positives that were missed in Kenya was Coombs positive, whilst one of the two that was missed in Spain was SAT positive. Problems around repeatability are likely to be minimised through the use of a glossy white tile, testing only a limited number of sera each time, adequate training of technicians to recognise all agglutination patterns, and the use of good quality controls [25]. The measurement of RBT titres using serial dilution, and particularly evidence of rising titres on repeat testing, can provide better evidence of infection when combined with clinical presentation and adequate history taking [25,57]. Confirmation of agglutination results with a second test would also contribute to improved diagnostic accuracy. The LFA used in this study provides a simple and inexpensive option, and was found to have good test performance when manufactured by the Royal Tropical Institute, Netherlands [30]. These tests can distinguish between IgM and IgG antibodies, and can therefore also contribute to the clinical assessment of the stage of infection [59,60].

We do not have additional aetiological information for those patients enrolled in this study who appear to have had false positive brucellosis diagnoses, and an important question remains regarding the cause of their illnesses. In addition to HIV, malaria and typhoid, for which many recruited participants were also tested by participating health centres, a wide range of infectious causes of febrile illness are known to be prevalent in the study area. These include *Coxiella burnetii* [61,62], *Leptospira* spp. [63], *Rickettsia* spp. [64], and Chikungunya virus [65]. To our knowledge, neither health facility routinely tested for any of these pathogens at the time of the study, and while rapid tests are available for some, these often have poor diagnostic performance [66] and have not been widely adopted [67]. The demonstration that *Brucella* may be less common than anticipated in this setting therefore increases the diagnostic challenge for clinicians. Improved diagnostic services, including the development and validation of point of care tests and testing strategies, and the development of diagnostic and management algorithms for patients presenting with febrile illness, are urgently needed in this setting, as in many others in the region [19,54].

### Summary

The findings from this study strongly suggest that human brucellosis is being over-diagnosed in a mixed farming area of western Kenya. We expect that this is contributing to the over use of antibiotics, and has important economic impacts on affected patients. The FBAT used in government facilities throughout Kenya appears to have very poor diagnostic specificity and should be phased out. Further studies are needed to assess alternative diagnostic tests and testing strategies that can replace the FBAT in health facilities in the region. However, in the short term, and while awaiting the development of these alternatives, published recommendations and the results from this study suggest that the standard RBT, with antigen sourced from established sources with high standards of quality control, would provide a better alternative than the FBAT for the laboratory diagnosis of human brucellosis.

## Supporting information

S1 Checklist STROBE checklist (PDF)

## References

[1] McDermott JJ, Arimi SM. Brucellosis in sub-Saharan Africa: epidemiology, control and impact. Vet Microbiol. 2002;90: 111–134. 12414138 10.1016/s0378-1135(02)00249-3

[2] McDermott J, Grace D, Zinsstag J. Economics of brucellosis impact and control in low-income countries. Rev Sci Tech. 2013;32: 249–261. 23837382 10.20506/rst.32.1.2197

[3] Godfroid J, Cloeckaert A, Liautard J-P, Kohler S, Fretin D, Walravens K, et al From the discovery of the Malta fever's agent to the discovery of a marine mammal reservoir, brucellosis has continuously been a re-emerging zoonosis. Vet Res. 2005;36: 313–326. doi: 10.1051/vetres:2005003 15845228 10.1051/vetres:2005003

[4] Corbel MJ. Brucellosis: an overview. Emerging Infect Dis. 1997;3: 213–221. doi: 10.3201/eid0302.970219 9204307 10.3201/eid0302.970219 PMC2627605

[5] Kadohira M, McDermott JJ, Shoukri MM, Kyule MN. Variations in the prevalence of antibody to brucella infection in cattle by farm, area and district in Kenya. Epidemiol Infect. Cambridge University Press; 1997;118: 35–41. 9042033 10.1017/s0950268896007005 PMC2808770

[6] Muendo EN, Mbatha PM, Macharia J, Abdoel TH, Janszen PV, Pastoor R, et al Infection of cattle in Kenya with Brucella abortus biovar 3 and Brucella melitensis biovar 1 genotypes. Trop Anim Health Prod. Springer Netherlands; 2012;44: 17–20. doi: 10.1007/s11250-011-9899-9 21660647 10.1007/s11250-011-9899-9

[7] Philpott M, Auko O. Caprine brucellosis in Kenya. Br Vet J. 1972;128: 642–651. 4647703 10.1016/s0007-1935(17)36637-x

[8] Young EJ. An overview of human brucellosis. Clin Infect Dis. 1995;21: 283–290. 8562733 10.1093/clinids/21.2.283

[9] Franco MP, Mulder M, Gilman RH, Smits HL. Human brucellosis. Lancet Infect Dis. 2007;7: 775–786. doi: 10.1016/S1473-3099(07)70286-4 18045560 10.1016/S1473-3099(07)70286-4

[10] Dean AS, Crump L, Greter H, Hattendorf J, Schelling E, Zinsstag J. Clinical manifestations of human brucellosis: a systematic review and meta-analysis. PLoS Negl Trop Dis. 2012;6: e1929 doi: 10.1371/journal.pntd.0001929 23236528 10.1371/journal.pntd.0001929 PMC3516581

[11] Ariza J. Brucellosis: an update. The perspective from the Mediterranean basin. Reviews in Medical Microbiology. 1999;10: 125–135.

[12] Buzgan T, Karahocagil MK, Irmak H, Baran AI, Karsen H, Evirgen O, et al Clinical manifestations and complications in 1028 cases of brucellosis: a retrospective evaluation and review of the literature. Int J Infect Dis. 2010;14: e469–78. doi: 10.1016/j.ijid.2009.06.031 19910232 10.1016/j.ijid.2009.06.031

[13] Ariza J, Bosilkovski M, Cascio A, Colmenero JD, Corbel MJ, Falagas ME, et al Perspectives for the treatment of brucellosis in the 21st century: the Ioannina recommendations. PLoS Med. 2007;4: e317 doi: 10.1371/journal.pmed.0040317 18162038 10.1371/journal.pmed.0040317 PMC2222927

[14] Zinsstag J, Schelling E, Roth F, Bonfoh B, de Savigny D, Tanner M. Human benefits of animal interventions for zoonosis control. Emerging Infect Dis. 2007;13: 527–531. doi: 10.3201/eid1304.060381 17553265 10.3201/eid1304.060381 PMC2725951

[15] Colmenero JD, Cabrera FP, Hernadez S, Reguera JM, Pinedo A, Clavero AMC. Repercusión socio-económica de la brucelosis humana. Rev Clinica Espanola. 1989;185: 55–63.

[16] Corbel MJ. Brucellosis in Humans and Animals. WHO; 2006 pp. 1–90.

[17] Ducrotoy M, Bertu WJ, Matope G, Cadmus S, Conde-Álvarez R, Gusi AM, et al Brucellosis in Sub-Saharan Africa: Current challenges for management, diagnosis and control. Acta Trop. 2015. 10.1016/j.actatropica.2015.10.023 26551794

[18] Spink WW. The diagnosis of human brucellosis. The nature of brucellosis. The University of Minnesota Press; 1956 pp. 191–215.

[19] Crump JA, Morrissey AB, Nicholson WL, Massung RF, Stoddard RA, Galloway RL, et al Etiology of severe non-malaria febrile illness in Northern Tanzania: a prospective cohort study. PLoS Negl Trop Dis. 2013;7: e2324 doi: 10.1371/journal.pntd.0002324 23875053 10.1371/journal.pntd.0002324 PMC3715424

[20] Marcotty T, Matthys F, Godfroid J, Rigouts L, Ameni G, Gey van Pittius N, et al Zoonotic tuberculosis and brucellosis in Africa: neglected zoonoses or minor public-health issues? The outcomes of a multi-disciplinary workshop. Ann Trop Med Parasitol. 2009;103: 401–411. doi: 10.1179/136485909X451771 19583911 10.1179/136485909X451771

[21] Omer MK, Assefaw T, Skjerve E, Tekleghiorghis T, Woldehiwet Z. Prevalence of antibodies to Brucella spp. and risk factors related to high-risk occupational groups in Eritrea. Epidemiol Infect. 2002;129: 85–91. 12211600 10.1017/s0950268802007215 PMC2869878

[22] Kunda J, Fitzpatrick J, Kazwala R, French NP, Shirima G, Macmillan A, et al Health-seeking behaviour of human brucellosis cases in rural Tanzania. BMC Public Health. 2007;7: 315 doi: 10.1186/1471-2458-7-315 17980046 10.1186/1471-2458-7-315 PMC2186323

[23] Pappas G, Papadimitriou P, Akritidis N, Christou L, Tsianos EV. The new global map of human brucellosis. Lancet Infect Dis. 2006;6: 91–99. doi: 10.1016/S1473-3099(06)70382-6 16439329 10.1016/S1473-3099(06)70382-6

[24] Diaz R, Moriyón I. Laboratory techniques in the diagnosis of human brucellosis. In: Young EJ, Corbel MJ, editors. Brucellosis 1989 pp. 73–83.

[25] Díaz R, Casanova A, Ariza J, Moriyón I. The Rose Bengal Test in human brucellosis: a neglected test for the diagnosis of a neglected disease. PLoS Negl Trop Dis. 2011;5: e950 doi: 10.1371/journal.pntd.0000950 21526218 10.1371/journal.pntd.0000950 PMC3079581

[26] Ariza J, Pellicer T, Pallares R, Foz A, Gudiol F. Specific antibody profile in human brucellosis. Clin Infect Dis. 1992;14: 131–140. 1571417 10.1093/clinids/14.1.131

[27] Gazapo E, Gonzalez Lahoz J, Subiza JL, Baquero M, Gil J, la Concha de EG. Changes in IgM and IgG antibody concentrations in brucellosis over time: importance for diagnosis and follow-up. J Infect Dis. 1989;159: 219–225. 2915152 10.1093/infdis/159.2.219

[28] Wilson MM, Merrifield EVO. The antiglobulin (Coombs) test in brucellosis. Lancet. 1951;2: 913–914. 14881511 10.1016/s0140-6736(51)91875-2

[29] Smits HL, Basahi MA, Diaz R, Marrodan T, Douglas JT, Rocha A, et al Development and evaluation of a rapid dipstick assay for serodiagnosis of acute human brucellosis. J Clin Microbiol. 1999;37: 4179–4182. 10565959 10.1128/jcm.37.12.4179-4182.1999 PMC85920

[30] Smits HL, Abdoel TH, Solera J, Clavijo E, Díaz R. Immunochromatographic Brucella-specific immunoglobulin M and G lateral flow assays for rapid serodiagnosis of human brucellosis. Clin Diagn Lab Immunol. 2003;10: 1141–1146. doi: 10.1128/CDLI.10.6.1141-1146.2003 14607880 10.1128/CDLI.10.6.1141-1146.2003 PMC262433

[31] Araj GF. Update on laboratory diagnosis of human brucellosis. Int J Antimicrob Agents. 2010;36 Suppl 1: S12–7. 20692128 10.1016/j.ijantimicag.2010.06.014

[32] Al-Dahouk S, Tomaso H, Nöckler K, Neubauer H, Frangoulidis D. Laboratory-based diagnosis of brucellosis—a review of the literature. Part II: serological tests for brucellosis. Clin Lab. 2003;49: 577–589. 14651329

[33] Young EJ. Serologic diagnosis of human brucellosis: analysis of 214 cases by agglutination tests and review of the literature. Rev Infect Dis. 1991;13: 359–372. 1866536 10.1093/clinids/13.3.359

[34] Dean AS, Crump L, Greter H, Schelling E, Zinsstag J. Global burden of human brucellosis: a systematic review of disease frequency. PLoS Negl Trop Dis. 2012;6: e1865 doi: 10.1371/journal.pntd.0001865 23145195 10.1371/journal.pntd.0001865 PMC3493380

[35] de Clare Bronsvoort BM, Thumbi SM, Poole EJ, Kiara H, Auguet OT, Handel IG, et al Design and descriptive epidemiology of the Infectious Diseases of East African Livestock (IDEAL) project, a longitudinal calf cohort study in western Kenya. BMC Vet Res. 2013;9: 171 doi: 10.1186/1746-6148-9-171 24000820 10.1186/1746-6148-9-171 PMC3847666

[36] Spink WW. The natural course of brucellosis. The nature of brucellosis. The University of Minnesota Press; 1956 pp. 145–170.

[37] OIE. Bovine Brucellosis. Manual of Diagnostic Tests and Vaccines for Terrestrial Animals. 2009. pp. 1–35.

[38] Blasco JM, Garin-Bastuji B, Marin CM, Gerbier G, Fanlo J, Jiménez de Bagués MP, et al Efficacy of different Rose Bengal and complement fixation antigens for the diagnosis of Brucella melitensis infection in sheep and goats. Vet Rec. 1994;134: 415–420. 8036772 10.1136/vr.134.16.415

[39] Renoux G, Plommet M, Philippon A. Microreactions d'agglutination et de fixation du complement pour le diagnostic des brucellosis. Ann Rech Vet. 1971;2: 263–269.

[40] Beaton CP, Forsyth JR. A micromethod for agglutination and antiglobulin tests for antibody to Brucella abortus. J Biol Stand. 1984;12: 271–275. 6434543 10.1016/s0092-1157(84)80006-2

[41] Harrell FE. Regression modeling strategies, with applications to linear models, survival analysis and logistic regression. Springer; 2001 pp. 1–582.

[42] Diaz R, Ariza J, Alberola I, Casanova A, Rubio MF. Secondary serological response of patients with chronic hepatosplenic suppurative brucellosis. Clin Vaccine Immunol. 2006;13: 1190–1196. doi: 10.1128/CVI.00086-06 16943345 10.1128/CVI.00086-06 PMC1656543

[43] Woods CR. False-Positive Results for Immunoglobulin M Serologic Results: Explanations and Examples. J Pediatric Infect Dis Soc. 2013;2: 87–90. doi: 10.1093/jpids/pis133 26619450 10.1093/jpids/pis133

[44] Swai ES, Schoonman L. Human brucellosis: seroprevalence and risk factors related to high risk occupational groups in Tanga Municipality, Tanzania. Zoonoses Public Health. 2009;56: 183–187. doi: 10.1111/j.1863-2378.2008.01175.x 18811674 10.1111/j.1863-2378.2008.01175.x

[45] Goldstein BP. Resistance to rifampicin: a review. J Antibiot. 2014;67: 625–630. doi: 10.1038/ja.2014.107 25118103 10.1038/ja.2014.107

[46] Brink M. Brucellosis in Kenya: Epidemiology and Human Burden of a Neglected Zoonotic Disease. MSc Thesis, University of Uppsala. 2013.

[47] Osoro EM, Munyua P, Omulo S, Ogola E, Ade F, Mbatha P, et al Strong Association Between Human and Animal Brucella Seropositivity in a Linked Study in Kenya, 2012–2013. Am J Trop Med Hyg. 2015;93: 224–231. doi: 10.4269/ajtmh.15-0113 26101275 10.4269/ajtmh.15-0113 PMC4530738

[48] Ducrotoy MJ, Conde-Álvarez R, Blasco JM, Moriyón I. A review of the basis of the immunological diagnosis of ruminant brucellosis. Vet Immunol Immunopathol. 2016;171: 81–102. doi: 10.1016/j.vetimm.2016.02.002 26964721 10.1016/j.vetimm.2016.02.002

[49] Wattam AR, Inzana TJ, Williams KP, Mane SP, Shukla M, Almeida NF, et al Comparative genomics of early-diverging Brucella strains reveals a novel lipopolysaccharide biosynthesis pathway. MBio. 2012;3: e00246–11. 10.1128/mBio.00388-12 PMC3509431 23131829

[50] Chipwaza B, Mhamphi GG, Ngatunga SD, Selemani M, Amuri M, Mugasa JP, et al Prevalence of bacterial febrile illnesses in children in Kilosa district, Tanzania. PLoS Negl Trop Dis. 2015;9: e0003750 doi: 10.1371/journal.pntd.0003750 25955522 10.1371/journal.pntd.0003750 PMC4425467

[51] Huddleson IF. Laboratory diagnosis of brucellosis Brucellosis in man and animals. The Commonwealth Fund; 1943 pp. 214–237.

[52] John K, Kazwala R, Mfinanga GS. Knowledge of causes, clinical features and diagnosis of common zoonoses among medical practitioners in Tanzania. BMC Infect Dis. 2008;8: 162 doi: 10.1186/1471-2334-8-162 19046464 10.1186/1471-2334-8-162 PMC2611996

[53] Zhang HL, Mnzava KW, Mitchell ST, Melubo ML, Kibona TJ, Cleaveland S, et al Mixed Methods Survey of Zoonotic Disease Awareness and Practice among Animal and Human Healthcare Providers in Moshi, Tanzania. PLoS Negl Trop Dis. 2016;10:10.1371/journal.pntd.0004476 PMC4778930 26943334

[54] D'Acremont V, Kilowoko M, Kyungu E, Philipina S, Sangu W, Kahama-Maro J, et al Beyond malaria—causes of fever in outpatient Tanzanian children. N Engl J Med. 2014;370: 809–817. doi: 10.1056/NEJMoa1214482 24571753 10.1056/NEJMoa1214482

[55] Mantur BG, Biradar MS, Bidri RC, Mulimani MS, Veerappa, Kariholu P, et al Protean clinical manifestations and diagnostic challenges of human brucellosis in adults: 16 years' experience in an endemic area. J Med Microbiol. 2006;55: 897–903. doi: 10.1099/jmm.0.46097-0 16772417 10.1099/jmm.0.46097-0

[56] Oomen LJ, Waghela S. The rose bengal plate test in human brucellosis. Trop Geogr Med. 1974;26: 300–302. 4439467

[57] Mantur BG, Amarnath SK, Patil GA, Desai AS. Clinical utility of a quantitative Rose Bengal slide agglutination test in the diagnosis of human brucellosis in an endemic region. Clin Lab. 2014;60: 533–541. 24779287 10.7754/clin.lab.2013.121120

[58] Maichomo MW, McDermott JJ, Arimi SM, Gathura PB. Assessment of the Rose-Bengal plate test for the diagnosis of human brucellosis in health facilities in Narok district, Kenya. East Afr Med J. 1998;75: 219–222. 9745838

[59] Mizanbayeva S, Smits HL, Zhalilova K, Abdoel TH, Kozakov S, Ospanov KS, et al The evaluation of a user-friendly lateral flow assay for the serodiagnosis of human brucellosis in Kazakhstan. Diagn Microbiol Infect Dis. 2009;65: 14–20. doi: 10.1016/j.diagmicrobio.2009.05.002 19679230 10.1016/j.diagmicrobio.2009.05.002

[60] Roushan MRH, Amin MJS, Abdoel TH, Smits HL. Application of a user-friendly Brucella-specific IgM and IgG antibody assay for the rapid confirmation of Rose Bengal-positive patients in a hospital in Iran. Trans R Soc Trop Med Hyg. 2005;99: 744–750. doi: 10.1016/j.trstmh.2005.02.009 16095642 10.1016/j.trstmh.2005.02.009

[61] Wardrop NA, Thomas LF, Cook EAJ, de Glanville WA, Atkinson PM, Wamae CN, et al The Sero-epidemiology of Coxiella burnetii in Humans and Cattle, Western Kenya: Evidence from a Cross-Sectional Study. PLoS Negl Trop Dis. 2016;10: e0005032 doi: 10.1371/journal.pntd.0005032 27716804 10.1371/journal.pntd.0005032 PMC5055308

[62] Knobel DL, Maina AN, Cutler SJ, Ogola E, Feikin DR, Junghae M, et al Coxiella burnetii in humans, domestic ruminants, and ticks in rural western Kenya. Am J Trop Med Hyg. 2013;88: 513–518. doi: 10.4269/ajtmh.12-0169 23382156 10.4269/ajtmh.12-0169 PMC3592534

[63] Cook EAJ, de Glanville WA, Thomas LF, Kariuki S, Bronsvoort BM de C, Fèvre EM. Risk factors for leptospirosis seropositivity in slaughterhouse workers in western Kenya. Occup Environ Med. 2016:10.1136/oemed-2016-103895 PMC5520261 27913579

[64] Maina AN, Knobel DL, Jiang J, Halliday J, Feikin DR, Cleaveland S, et al Rickettsia felis infection in febrile patients, western Kenya, 2007–2010. Emerging Infect Dis. 2012;18: 328–331. doi: 10.3201/eid1802.111372 22304807 10.3201/eid1802.111372 PMC3310467

[65] Mease LE, Coldren RL, Musila LA, Prosser T, Ogolla F, Ofula VO, et al Seroprevalence and distribution of arboviral infections among rural Kenyan adults: a cross-sectional study. Virol J. 2011;8: 371 doi: 10.1186/1743-422X-8-371 21794131 10.1186/1743-422X-8-371 PMC3161961

[66] Bajani MD, Ashford DA, Bragg SL, Woods CW, Aye T, Spiegel RA, et al Evaluation of four commercially available rapid serologic tests for diagnosis of leptospirosis. J Clin Microbiol. 2003;41: 803–809. doi: 10.1128/JCM.41.2.803-809.2003 12574287 10.1128/JCM.41.2.803-809.2003 PMC149700

[67] Picardeau M, Bertherat E, Jancloes M, Skouloudis AN, Durski K, Hartskeerl RA. Rapid tests for diagnosis of leptospirosis: current tools and emerging technologies. Diagn Microbiol Infect Dis. 2014;78: 1–8. doi: 10.1016/j.diagmicrobio.2013.09.012 24207075 10.1016/j.diagmicrobio.2013.09.012

